# Supplementary material for: Incidence and Predictors of Permanent Pacemaker Implantation after Transcatheter Aortic Valve Procedures: Data of The Netherlands Heart Registration (NHR)
Source: J Clin Med. 2022 Jan 23;11(3):560. doi: 10.3390/jcm11030560 (PMC8836997; doi:10.3390/jcm11030560)
Supplement: Supplementary file 1 [file jcm-11-00560-s001.zip › jcm-1563382-supplementary.pdf]

Table S1. Univariable analysis of 30-day permanent pacemaker (PM) implantation after transcatheter aortic valve implantation (TAVI).

**Variables**	**OR (95% CI)**	***P* value**
Male sex	0.77 (0.67 - 0.89)	<0.01
Age (years)	1.01 (1.00 - 1.02)	0.13
Weight (kg)	1.01 (1.00 - 1.01)	<0.01
Creatinine serum level (for 100 µmol/L)	1.15 (1.05 - 1.25)	<0.01
Diabetes mellitus	1.07 (0.92 - 1.25)	0.38
Left ventricular ejection fraction (%)	1.00 (1.00 - 1.01)	0.71
Systolic pulmonary pressure (mmHg)	1.00 (1.00 - 1.01)	0.43
History of lung disease	1.00 (0.84 - 1.18)	0.99
Peripheral vascular disease	0.96 (0.81 - 1.14)	0.67
Previous cardiac surgery	0.87 (0.73 - 1.04)	0.12
Recent myocardial infarction	0.80 (0.46 - 1.40)	0.44
Dialysis	1.47 (0.86 - 2.53)	0.16
Euroscore II (%)	0.99 (0.98 - 1.01)	0.40
Previous aortic valve surgery	0.42 (0.26 - 0.67)	<0.01
Transfemoral TAVI access	1.35 (1.13 - 1.61)	<0.01
Pre-dilatation valve	0.90 (0.78 - 1.04)	0.15
Post-dilatation valve	1.62 (1.37 - 1.93)	<0.01

OR = odds ratio; CI = confidence interval, TAVI = Transcatheter Aortic Valve Implantation.

Table S2. Backward Stepwise Elimination of 30-day permanent pacemaker (PM) implantation after transcatheter aortic valve implantation.

**Variables**	**OR (95% CI)**	***P* value**
Male sex	0.82 (0.70 – 0.95)	<0.01
Age (years)	1.01 (1.00 – 1.02)	0.04
Weight (kg)	1.01 (1.00 – 1.01)	0.05
Creatinine serum level (for 100 µmol/L)	1.13 (1.04 – 1.23)	<0.01
Previous aortic valve surgery	0.42 (0.26 – 0.67)	<0.01
Transfemoral TAVI access	1.33 (1.11 – 1.60)	<0.01
Post-dilatation valve	1.61 (1.35 – 1.91)	<0.01

OR = odds ratio; CI = confidence interval, TAVI = Transcatheter Aortic Valve Implantation.

Addendum S1. Page containing all author's details

Justine M. Ravaux MD

Corresponding Author
Mail: jmravaux@hotmail.com
Adress:
Department of Cardio-Thoracic Surgery,
Heart and Vascular Centre,
Maastricht University Medical Centre (MUMC),
P. Debyelaan, 25,
6202 AZ
Maastricht, The Netherlands
Phone: +32(0) 472 59 73 59
Short CV: @MafaldaRavaux

Sander MJ Van Kuijk PhD

Mail: sander.van.kuijk@mumc.nl
Adress:
Department of Cardio-Thoracic Surgery,
Heart and Vascular Centre,
Maastricht University Medical Centre (MUMC),
P. Debyelaan, 25,
6202 AZ
Maastricht, The Netherlands
Phone *:* + 31(0) 433877095
Short CV : https://www.mumc.nl/research/kemta/ons-team

Michele Di Mauro MD, PhD, MSc Biostat

Mail: mdimauro1973@gmail.com
Adress:
Department of Cardio-Thoracic Surgery,
Heart and Vascular Centre,
Maastricht University Medical Centre (MUMC),
P. Debyelaan, 25,
6202 AZ
Maastricht, The Netherlands
Phone *:* + 31(0) 433877095
Short CV: https://it.linkedin.com/in/michele-di-mauro-a3a45b102

Kevin Vernooy MD, PhD

Mail: kevin.vernooy@mumc.nl
Adress :
Department of Cardio-Thoracic Surgery,
Heart and Vascular Centre,
Maastricht University Medical Centre (MUMC),
P. Debyelaan, 25,
6202 AZ
Maastricht, The Netherlands
Phone *:* + 31(0) 433877095
Short CV: https://www.mumc.nl/specialisten/vernooy

Elham Bidar MD, PhD

Mail: elham.bidar@mumc.nl
Adress :
Department of Cardio-Thoracic Surgery,
Heart and Vascular Centre,
Maastricht University Medical Centre (MUMC),
P. Debyelaan, 25,
6202 AZ
Maastricht, The Netherlands
Phone : + 31(0) 433877095
Short CV: https://www.mumc.nl/specialisten/bidar-0

Arnoud W Van't Hof MD, PhD

Mail: arnoud.vant.hof@mumc.nl
Adress :
Department of Cardio-Thoracic Surgery,
Heart and Vascular Centre,
Maastricht University Medical Centre (MUMC),
P. Debyelaan, 25,
6202 AZ
Maastricht, The Netherlands
Phone : + 31(0) 433877095
Short CV: https://www.mumc.nl/specialisten/hof-van-t

Leo Veenstra MD

Mail: l.veenstra@mumc.nl
Adress :
Department of Cardio-Thoracic Surgery,
Heart and Vascular Centre,
Maastricht University Medical Centre (MUMC),
P. Debyelaan, 25,
6202 AZ
Maastricht, The Netherlands
Phone : + 31(0) 433877095
Short CV : https://www.zuyderland.nl/medewerkers/l-veenstra/

Suzanne Kats MD, PhD

Mail: suzanne.kats@mumc.nl
Adress :
Department of Cardio-Thoracic Surgery,
Heart and Vascular Centre,
Maastricht University Medical Centre (MUMC),
P. Debyelaan, 25,
6202 AZ
Maastricht, The Netherlands
Phone : + 31(0) 433877095
Short CV: https://nl.linkedin.com/in/suzanne-kats-md-phd-410b42183

Saskia Houterman , PhD

Mail: saskia.houterman@nederlandsehartregistratie.nl
Adress :
Nederlandse Hart Registratie, locatie Eindhoven,
Postbus 1350 – 5602
ZA Eindhoven
The Netherlands

Phone : + 31(0) 88 2200 929
Short CV: https://nl.linkedin.com/in/saskia-houterman-8a1a0760

Jos G Maessen MD, PhD

Mail: j.g.maessen@mumc.nl
Adress :
Department of Cardio-Thoracic Surgery,
Heart and Vascular Centre,
Maastricht University Medical Centre (MUMC),
P. Debyelaan, 25,
6202 AZ
Maastricht, The Netherlands
Phone : + 31(0) 433877095
Short CV: https://www.mumc.nl/specialisten/maessen

Roberto Lorusso MD, PhD

Mail: roberto.lorussobs@gmail.com
Adress :
Department of Cardio-Thoracic Surgery,
Heart and Vascular Centre,
Maastricht University Medical Centre (MUMC),
P. Debyelaan, 25,
6202 AZ
Maastricht, The Netherlands
Phone : + 31(0) 433877095
Short CV : https://www.ctsnet.org/home/rlorusso

Addendum S2. Definitions of baseline characteristics

Diabetes mellitus : Diabetes mellitus diagnosed prior to intervention is characterized by chronic hyperglycaemia where one of the following criteria is demonstrated:

- Fasting plasma glucose level ≥ 7.0 mmol/L (≥ 126 mg/dL)
- Plasma glucose ≥ 11.1 mmol/L (≥ 200 mg/dL) two hours after taking 75g oral glucose as in the glucose tolerance test (plasma glucose measurement should not be taken at the time of or shortly after a glucose infusion)
- Symptoms of hyperglycaemia and casually measured plasma glucose ≥ 11.1 mmol/L (≥ 200 mg/dL) (plasma glucose measurement should not take place at the time of or shortly after a glucose infusion)
- Glycosylated Hemoglobin (HbA1c) ≥ 6.5%

Systolic pulmonary pressure : Pulmonary artery systolic pressure in mmHg measured by an invasive pressure reading or estimated from an echocardiography preceding the current intervention. The exactly measured pressure value is preferably supplied. If there is only one descriptive value recorded of pulmonary hypertension (e.g. mag), the following pressures are recorded following the list below:

- Normal pressure = 25 mmHg
- Middle elevated pressure = 40 mmHg
- Severe Elevated Pressure = 60 mmHg

History of lung disease : long-term use of bronchodilatators or steroids because of lung disease.

Peripheral vascular disease : if one of more criteria below are present:

- Intermittent claudication
- Carotid occlusion or >50% stenosis
- Amputation due to arterial disease
- Previous or planned surgery on abdominal aorta, arteries of the limbs or carotids.
